# A Fatal Case of a Ruptured Posterior Communicating Artery Aneurysm in a Patient With Suspected Klippel-Feil Syndrome

**DOI:** 10.7759/cureus.96575

**Published:** 2025-11-11

**Authors:** Jason Dagoon, Se Jong Choi, James Park, Chul Chae

**Affiliations:** 1 Radiology, California University of Science and Medicine, Colton, USA; 2 Radiology, Arrowhead Regional Medical Center, Colton, USA; 3 Internal Medicine, California University of Science and Medicine, Colton, USA; 4 Medical Imaging, Arrowhead Regional Medical Center, Colton, USA

**Keywords:** aneurysmal subarachnoid hemorrhage, cervical vertebrae fusion, complicated subarachnoid hemorrhage, incidental aneurysm, incidental radiological finding, internal carotid artery aneurysm, klippel feil, klippel feil syndrome, posterior communicating artery (pcom)

## Abstract

Klippel-Feil syndrome (KFS) is a rare congenital disorder characterized by cervical vertebral fusion and often associated with multisystem anomalies. Although primarily skeletal, KFS has been linked to vascular abnormalities, including arterial dissections, agenesis, and aneurysms. We present the case of a 33-year-old previously healthy woman who presented with diffuse subarachnoid hemorrhage. Imaging revealed a ruptured 5.5 mm posterior communicating artery aneurysm arising from the left supraclinoid internal carotid artery, along with congenital cervical vertebral fusion consistent with undiagnosed KFS. The patient underwent emergency craniotomy with clip ligation complicated by intraoperative rupture and cerebral edema, ultimately requiring decompressive hemicraniectomy. Despite maximal surgical and medical management, she progressed to brain death. This case suggests a possible association between KFS and cerebrovascular anomalies and highlights the importance of considering vascular imaging in patients with KFS who present with acute neurological deterioration. Increased awareness of these potential complications may aid in earlier recognition and improved outcomes.

## Introduction

Klippel-Feil syndrome (KFS) is a rare congenital disorder characterized by cervical vertebral fusion [1,2]. Although classically associated with skeletal abnormalities [3-5], recent evidence highlights vascular manifestations that may have serious neurologic consequences [6-13]. While vertebral fusion may be incidentally detected on imaging, vascular anomalies are reported far less frequently, and their clinical significance remains under-recognized [2,6].

The estimated prevalence of KFS is approximately 1 in 40,000 live births, though large imaging studies suggest higher rates due to incidental detection in asymptomatic individuals [2,5]. Reported vascular abnormalities include arterial dissections, agenesis of the internal carotid artery, and vertebral or intracranial aneurysms, each described only in isolated cases [6-13]. These vascular manifestations may predispose patients to ischemic stroke or intracranial hemorrhage [6-13].

Although arterial dissections and agenesis of the internal carotid artery have been reported in KFS [6-10], aneurysmal subarachnoid hemorrhage remains exceedingly rare [11-13]. To our knowledge, only a handful of such cases exist. We report a fatal case of posterior communicating artery (PCOM) aneurysm rupture in a patient with radiographic features consistent with KFS, previously undiagnosed, underscoring the need to recognize potential cerebrovascular involvement in this congenital disorder.

## Case presentation

A 33-year-old previously healthy woman was found unresponsive at home by family members and transported to a local emergency department. On arrival, her Glasgow Coma Scale (GCS) score was 3T, with no eye opening, verbal response, or motor response. Pupils were initially miotic and nonreactive. She was intubated for airway protection, and naloxone was administered, but there was no improvement. Laboratory studies showed marked leukocytosis (WBC 27.2 K/µL), severe lactic acidosis (lactate 9.7 mmol/L), and hyperglycemia (glucose 245 mg/dL), findings considered reactive to severe intracranial hemorrhage (Table 1).

**Table 1:** Initial lab results

Parameter	Patient value	Reference range
WBC (K/µL)	27.2	4.0-11.0
Lactate (mmol/L)	9.7	0.5-2.0
Glucose (mg/dL)	245	70-99 (fasting)

**Figure 1:**
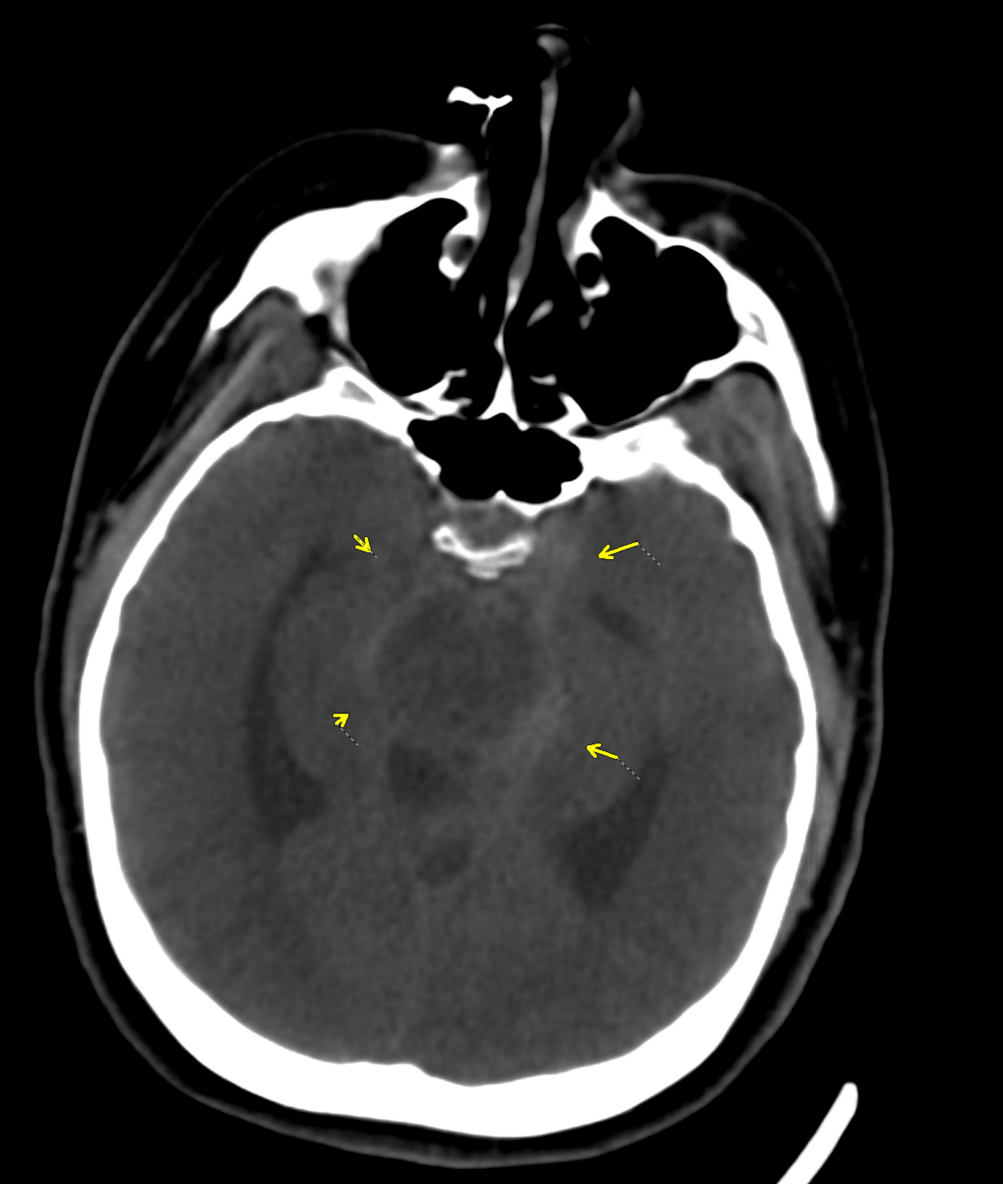
Axial non-contrast CT of the head showing diffuse subarachnoid hemorrhage (yellow arrows) within the basal cisterns. CT = computed tomography

A non-contrast computed tomography (CT) scan of the head revealed diffuse subarachnoid hemorrhage (Fisher grade 4) involving the basal cisterns, sylvian fissures, and anterior interhemispheric fissure, with early ventricular dilation and transtentorial herniation (Figure 1). The patient was transferred to our tertiary care center on an emergency basis.

**Figure 2:**
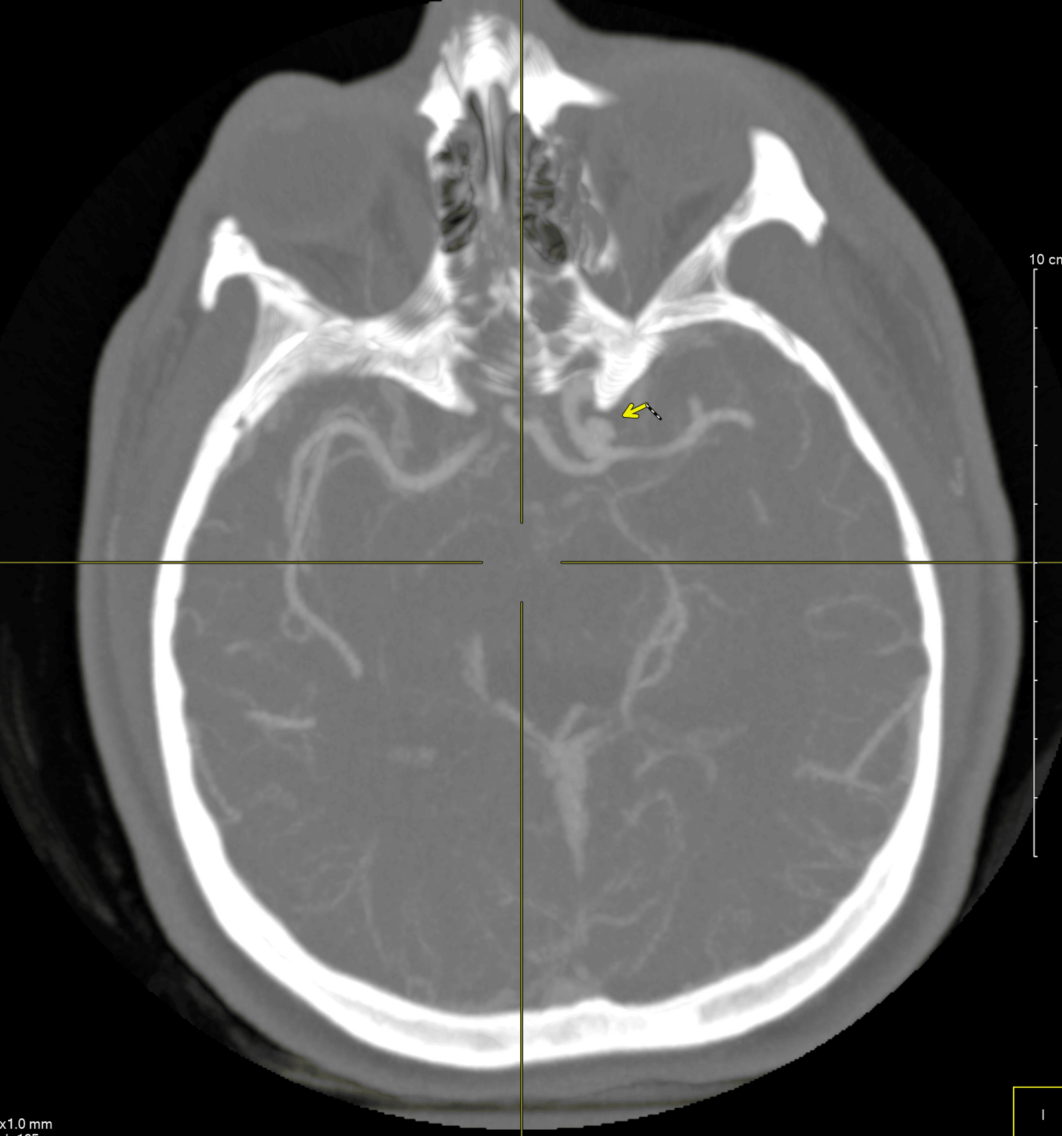
Axial CTA MIP reconstruction of the head demonstrating a left posterior communicating artery aneurysm (yellow arrow). MIP = maximum intensity projection; CTA = computed tomography angiography

**Figure 3:**
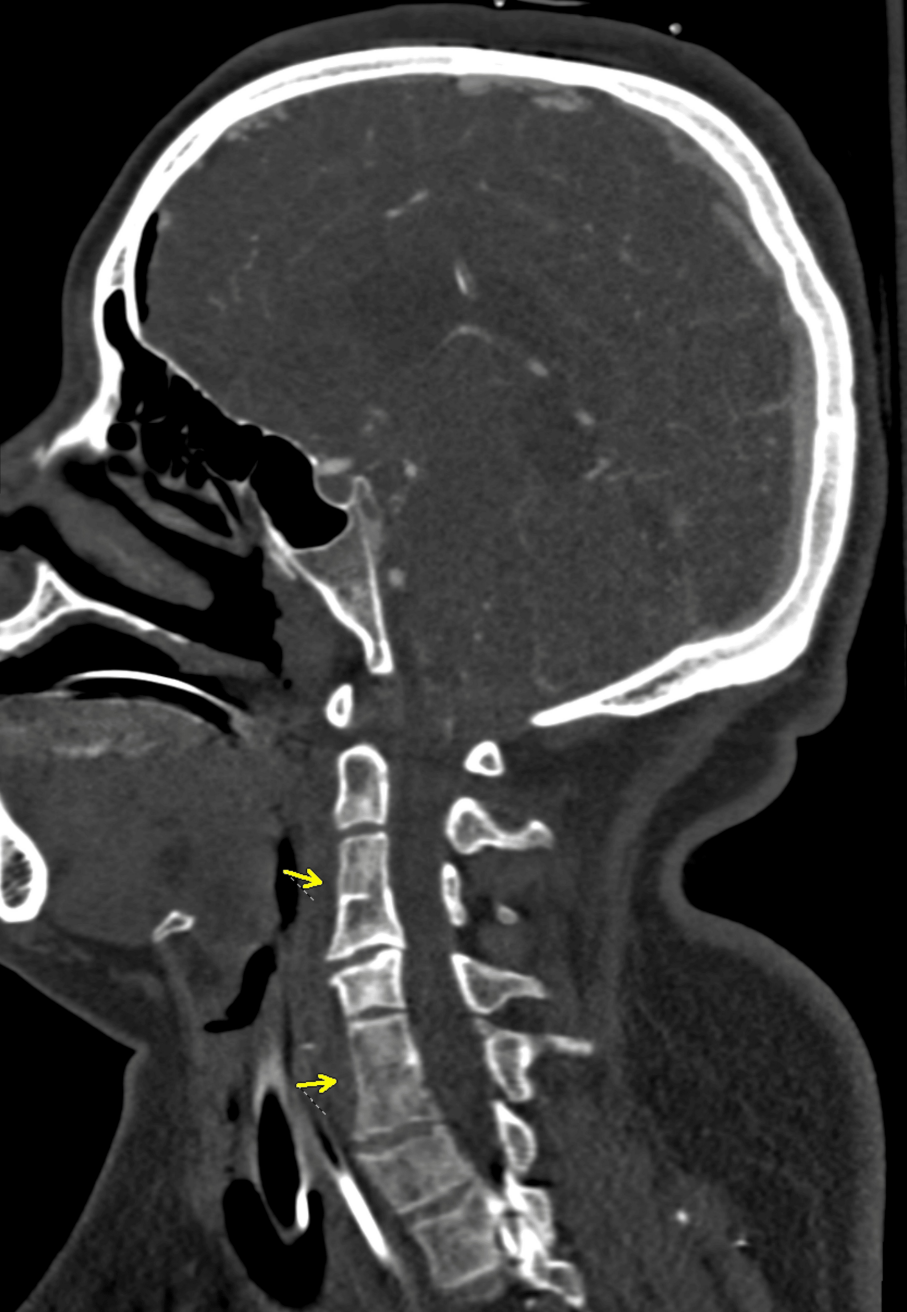
Sagittal CTA of the cervical spine demonstrating congenital C3-C4 and C6-C7 fusion, radiographic features suggestive of Klippel-Feil syndrome (yellow arrows). CTA = computed tomography angiography

Upon arrival, she remained comatose and hypotensive, requiring vasopressor support. An external ventricular drain (EVD) was placed, yielding an opening pressure >30 mmHg. Computed tomography angiography (CTA) demonstrated a 5.5 mm saccular aneurysm arising from the supraclinoid segment of the left internal carotid artery, consistent with a ruptured PCOM aneurysm (Figure 2). Incidentally, the CTA also showed congenital fusion of the C3-C4 and C6-C7 vertebrae, radiographic features suggestive of Klippel-Feil syndrome (Figure 3). The patient’s family denied any prior medical history or features of the classic KFS triad, suggesting an asymptomatic and previously undiagnosed case.

Given the aneurysm’s wide-neck morphology, endovascular coiling was not feasible. The patient underwent emergency left pterional craniotomy for clip ligation. During clipping, fragile blister-like outpouchings along the PCOM ruptured, necessitating temporary clipping and conversion to decompressive hemicraniectomy due to severe cerebral edema. Postoperatively, the patient’s pupils became bilaterally fixed and dilated, with absent brainstem reflexes. Despite maximal medical management, her neurologic and hemodynamic status deteriorated. Following discussion with her family, care was transitioned to comfort measures, and she was pronounced deceased later that evening.

## Discussion

This case highlights a rare convergence of a ruptured posterior communicating artery aneurysm and radiographic evidence of KFS. While the coexistence of cervical fusion and aneurysm rupture in this patient suggests a possible but unproven association, causality cannot be inferred from a single case. Nonetheless, this presentation underscores the importance of recognizing cerebrovascular manifestations that may accompany congenital skeletal anomalies.

Vascular abnormalities in KFS, although uncommon, have been increasingly documented. The majority involve extracranial vertebral arteries, including cases of arterial dissections and aneurysms likely related to altered cervical biomechanics and excessive C1-C2 motion [7-9,12]. Internal carotid artery agenesis and persistent embryonic arteries have also been reported [6,10,11]. In contrast, intracranial aneurysms, particularly involving the internal carotid artery or PCOM, remain exceedingly rare, with only isolated reports in the literature [12,13]. Our patient’s presentation, therefore, represents an unusual manifestation within the broader vascular spectrum of KFS.

The proposed link between vertebral fusion and vascular fragility may involve chronic mechanical strain, abnormal hemodynamic stress, or embryologic vessel wall dysgenesis [4,12]. Altered cervical motion and arterial tortuosity may generate localized turbulence and endothelial stress, leading to a predisposition for dissection or aneurysm formation. However, definitive mechanisms remain speculative, highlighting the need for further investigation.

The poor outcome in this case was primarily attributable to the patient’s initial Hunt-Hess grade V and Fisher grade 4 subarachnoid hemorrhage, both strongly associated with high mortality irrespective of underlying syndromic factors [14]. These grading scales provide important context for the clinical trajectory and emphasize that the fatal course was consistent with the severity of hemorrhage.

The intraoperative discovery of multiple fragile, blister-type aneurysms along the PCOM further complicated management. Such aneurysms are known for their thin walls, friability, and high rupture risk, often requiring complex microsurgical techniques-including temporary clipping or bypass-in addition to standard aneurysm ligation [15]. Their presence underscores the potential for challenging surgical management even beyond the syndromic context.

Given the expanding recognition of vascular manifestations in KFS, including dissections, agenesis, and aneurysms, clinicians should maintain a high index of suspicion for vascular pathology in symptomatic patients [6-13]. Advanced imaging with CT or MR angiography should be considered for individuals with KFS who present with acute neurologic deficits, headache, or signs of cerebrovascular insufficiency. For neurosurgeons, awareness of possible vascular anomalies may guide tailored operative planning, especially in cases involving blister aneurysms or abnormal cervical anatomy.

In summary, this case contributes to the limited literature describing an intracranial aneurysm rupture in KFS, an exceedingly rare occurrence compared with vertebral artery involvement [7-13]. It emphasizes cautious interpretation of potential associations, careful perioperative planning, and the value of vascular imaging in detecting occult anomalies in this congenital condition.

## Conclusions

Although KFS is primarily recognized for its skeletal manifestations, vascular complications, including dissections, agenesis, and aneurysms, have also been reported, albeit rarely. This case adds to that limited body of evidence. The poor outcome was largely attributable to the patient’s initial Hunt-Hess grade V and Fisher grade 4 subarachnoid hemorrhage, both of which independently predict high mortality. Clinicians should maintain a high index of suspicion for cerebrovascular anomalies in patients with KFS who present with acute neurological symptoms, as advanced vascular imaging (CTA or MRA) may be warranted. Though rare, such vascular events can be life-threatening and merit clinical vigilance.
